# Positive selection in cytochrome P450 genes is associated with gonad phenotype and mating strategy in social bees

**DOI:** 10.1038/s41598-023-32898-6

**Published:** 2023-04-11

**Authors:** Denyse Cavalcante Lago, Luísa Czamanski Nora, Martin Hasselmann, Klaus Hartfelder

**Affiliations:** 1 Department of Genetics, Ribeirão Preto School of Medicine (FMRP), University of São Paulo (USP), Ribeirão Preto, SP Brazil; 2 Department of Cell and Molecular Biology and Pathogenic Bioagents, Ribeirão Preto School of Medicine (FMRP), University of São Paulo (USP), Ribeirão Preto, SP Brazil; 3 Department of Livestock Population Genomics, Institute of Animal Science, University of Hohenheim, Stuttgart, Germany

**Keywords:** Developmental biology, Evolution, Genetics

## Abstract

The honey bee, *Apis mellifera* differs from all other social bees in its gonad phenotype and mating strategy. Honey bee queens and drones have tremendously enlarged gonads, and virgin queens mate with several males. In contrast, in all the other bees, the male and female gonads are small, and the females mate with only one or very few males, thus, suggesting an evolutionary and developmental link between gonad phenotype and mating strategy. RNA-seq comparisons of *A. mellifera* larval gonads revealed 870 genes as differentially expressed in queens *versus* workers and drones. Based on Gene Ontology enrichment we selected 45 genes for comparing the expression levels of their orthologs in the larval gonads of the bumble bee *Bombus terrestris* and the stingless bee, *Melipona quadrifasciata,* which revealed 24 genes as differentially represented. An evolutionary analysis of their orthologs in 13 solitary and social bee genomes revealed four genes with evidence of positive selection. Two of these encode cytochrome P450 proteins*,* and their gene trees indicated a lineage-specific evolution in the genus *Apis,* indicating that cytochrome P450 genes may be involved in the evolutionary association of polyandry and the exaggerated gonad phenotype in social bees.

## Introduction

The establishment of a highly eusocial form of life occurred independently in two closely related lineages of bees, the honey bees and the stingless bees^1,2^, in two lineages of wasps^3^, and in an ancestor common to all ants^4^. This highly eusocial lifestyle, characterized by large colonies composed of at least one highly reproductive queen and hundreds of subfertile or sterile workers, represents an irreversible major evolutionarily transition^5^, as the morphologically distinct queen and worker castes have become mutually completely dependent on one another. The development of such physical castes not only strengthened the queen’s reproductive monopoly, but also promoted division of labor and task specialization in the worker caste, which enabled the highly eusocial Hymenoptera to establish very large colony sizes.

This transition in the level of social organization was accompanied by a change in the queens’ mating strategy that occurred independently in several lineages of the ants, in two lineages of wasps (*Vespa* and *Vespula*) and in the honey bees (genus *Apis)*. While the females of the facultatively social or primitively eusocial bees and wasps generally mate with only a single male (monandry), the queens of several highly eusocial species are known to mate with more than two males (polyandry), which, in cases of extreme polyandry, can be 20 or more^6^. Polyandry has fitness consequences for the workers, as it diminishes the within-colony genetic relatedness, and thus, affects the structure of the genetic conflict over male production and sex ratio allocation^7,8^. As monandry is the ancestral condition^9^, the multiple mating systems and their associated male traits have drawn attention and generated controversial arguments about the adaptive significance of multiple mating in the highly eusocial Hymenoptera^10^. Among the eight hypotheses proposed for the evolution of polyandry in social insects^11^, the currently most accepted one is that it strengthens the colonies’ resistance against parasite or pathogen infections^12^. Polyandry, however, is not a simple categorical trait that stands in contrast with monandry, but there is a very strong skew in the number of recorded mating events^9,13^. Specifically, in only 0.2% of the known eusocial hymenopteran species the queen mates with more than three males, and mating frequencies with over 20 males are extremely rare^6^.

The Western honey bee, *Apis mellifera,* falls within the category of these extremely polyandrous species, with mean mating frequencies estimated from effective paternity calculations as varying between 13 and 20 (range 8–32)^14,15^. Hence, while on the one hand, *A. mellifera* is the best-established model organism for functional and structural aspects of the complex social organization in insects, the entire genus *Apis* strongly differs from all the other social bees in terms of its gonad morphology and mating behavior.

An adult honey bee queen has very large ovaries, each composed of 100–150 serial units (ovarioles), and referred to as a transgressive (exaggerated) ovary phenotype^16,17^, while the ovaries of an adult worker consist of only 2–12 ovarioles each^18,19^. In contrast, in all the other bees, the females have only 3–4 ovarioles per ovary^20,21^, including the primitive eusocial bumble bees (Bombini) and the highly eusocial stingless bees (Meliponini). Together with the solitary to facultatively eusocial orchid bees (Euglossini) and the honey bees (Apini), these four tribes form a monophyletic clade, the corbiculate bees^22^, and thus, the question is: why and how did the ovarian architecture of the honey bees become so different from all the other bees? Or put in other terms, what may have been the evolutionary conditions or drivers that favored the origin of the exaggerated ovary phenotype in the genus *Apis*?

To address the this question we decided to take an EvoDevo approach where we contrasted the larval gonad transcriptomes for three bee species that differ in gonad architecture and reproductive strategies within a well defined phylogenetic background. For this, we generated RNA-seq libraries of larval gonads of the bumbe bee, *Bombus terrestris*, and the stingless bee, *Melipona quadrifasciata,* for a comparativ transcriptomics approach against our prior RNA-seq data generated for the honey bee queen, worker, and drone larval gonads^23^.

## Results

### Comparative transcriptome analysis for honey bee, bumble bee, and stingless bee gonads

In terms of read numbers, the RNA-seq results for the larval gonads of the bumble bee *B. terrestris* (n = 3) and the stingless bee *M. quadrifasciata* (n = 3) were similar to those simultaneously obtained for the honey bee larval gonads (n = 3 each for workers, queens and drones)^23^, consisting of between 29,304 and 41,890 fragments per million, where more than 83% of these good quality reads could be mapped to the respective reference genomes of the three species. The alignment scores were high for *A. mellifera,* varying between 83 and 87.4%, and also for *B. terrestris* (79% to 86%), but were lower for *M. quadrifasciata* (60.8% to 63%). Hence, while the 15 RNA-seq libraries generated for this study had similar numbers in terms of good quality reads, they differed in their mapping rates. This difference is likely due to the better annotation quality of the honey bee^24^ and bumble bee genomes^25^, compared to that of the *M. quadrifasciata* genome^26^.

**Figure 1:**
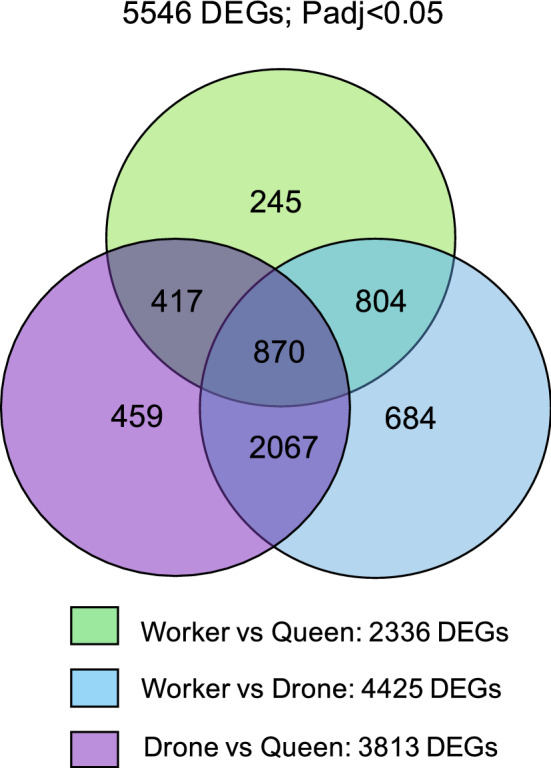
Venn-diagram representing differentially expressed genes (DEGs) in *Apis mellifera* queen, worker, and drone larval gonads. Three contrasts of comparisons were analyzed, resulting in a total of 5546 DEGs. A set of 870 DEGs is common to all contrasts.

As a first step in the cross-species comparison, we performed a differential expression analysis for the *A. mellifera* reads (DESeq2 based on normalized reads, *Padj* < 0.05), and here it is important to note that we used a less stringent cutoff level for differentially expressed genes (DEGs) than in our prior study on these honey bee larval gonad transcriptomes, where we had set the cutoff at *Padj* < 0.01^23^. The reason for adopting the less stringent DEG level in the current study was that for the subsequent hierarchical clustering against the bumble bee and stingless bee transcriptomes and the evolutionary analysis we needed a sufficiently large gene set of clear orthologs. This less stringent analysis revealed 5,546 genes as differentially expressed among the three types of honey bee larval gonads, including a central set of 870 genes that was common to all three contrasts (Fig. 1).

The 870 *Apis*-DEGs became the focal gene set for the comparative analysis with the bumble bee and the stingless bee larval gonads based on the reasoning that this DEGs set represents genes that are: (a) associated with the conservation of a high ovariole number in the queen caste, in contrast with the reduced ovariole number in the workers due to the massive programmed cell death in their larval gonads^17,23^, and (b) differentially expressed also between the two sexes, independent of caste in the female sex. A Gene Ontology (GO) analysis for these 870 DEGs returned 566 genes with GO information (Table S1), and enrichment was found for six categories.

For the bumble bee we obtained the orthologs of this gene set from the *B. terrestris* genome deposited in NCBI using the BLASTp tool. For *M. quadrifasciata,* the corresponding BLASTp searches were done in the Hymenoptera Genome Database (hymenopteragenome.org)^27^. In both cases we performed reciprocal BLAST searches against the honey bee genes. The RPKM counts (reads per kilobase per million mapped) for these orthologs were then retrieved from the respective RNA-seq data. We opted to use the RPKM normalization method because it eliminates the influence of gene length and variation in sequencing counts for comparisons between RNA-seq libraries^28^.

While our initial intention was to perform a hierarchical clustering analysis with all the 870 honey bee DEGs against their respective orthologs in the *B. terrestris* and *M. quadrifasciata* genomes, this was only possible for the comparison with the bumble bee, because many of the 870 honey bee DEGs showed unclear orthology relationships in the stingless bee genome*.* For *B. terrestris,* we obtained a total of 806 orthologs for the 870 *A. mellifera* DEGs, and the hierarchical clustering analysis performed in JMP Genomics software showed that the two species are clearly separated (Fig. S1) for this full gene set. This indicated already a possible connection between gonadal architecture and the mating system. Yet, including *M. quadrifasciata* in the analysis was fundamental, due to the fact that bumble bees differ from the honey bees and stingless bees with respect to their level of sociality (primitively vs. highly eusocial). Hence, we decided to perform the joint analysis for the three bee species with a reduced set of 45 DEGs that represent enriched GO categories (Table S2) and clear orthologies among the three species.

**Figure 2:**
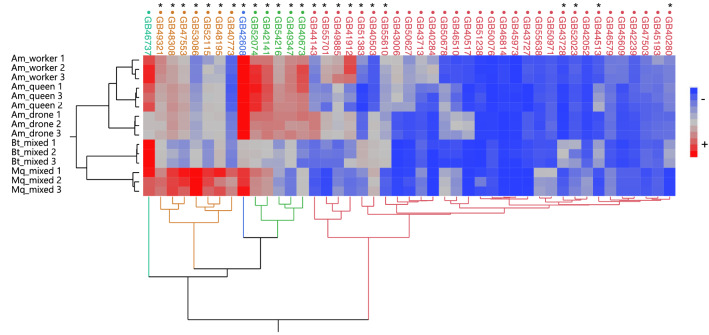
Hierarchical clustering analysis of the RPKM counts of 45 selected *A. mellifera* gonad DEGs and of their orthologs in the *Bombus terrestris* and *Melipona quadrifasciata* RNA-seq libraries. Each gene is identified by its honey bee gene ID. The red-blue scale represents relative RPKM counts. Asterisks denote the 24 DEGs with divergent RPKM levels in the gonad transriptomes of the three bee species.

As shown in Fig. 2, all nine *A. mellifera* libraries are grouped together and are well separated from the cluster composed by the respective *B. terrestris* and *M. quadrifasciata* orthologs. In this set of 45 genes, we could identify 24 that showed divergent RPKM levels for the three species, thus setting apart the gene expression patterns in the larval gonads of the two monandrous bees with an ancestral gonad morphology from the polyandrous honey bee with its derived, exaggerated gonad morphology. In Fig. 2, these 24 genes are marked with an asterisk, and Table S3 presents detailed descriptions.

A Principal Component Analysis (PCA) performed on the RPKM counts of these 45 genes in the 15 RNA-seq libraries produced a similar result (Fig. S2). The two first principal components (PC1 and PC2), which explain 96.25% of the total sample variance, not only separated the three species, but also placed the two highly eusocial species close to each other and clearly separated from the primitively eusocial bumble bee. This indicates that the factor “level of sociality” needs to be taken into account in transcriptome comparisons across social bees.

### Evolutionary analysis of gonad development-related genes in Apoidea

We next retrieved the nucleotide and amino acid sequences of the respective orthologs for the 24 divergently represented genes shown in Fig. 2 in the genomes of 13 bee species for which annotated sequences were available (Table S4), and we tested these for evidence of positive (diversifying) selection using the Branch-site Unrestricted Statistical Test for Episodic Diversification (BUSTED)^29^. This test assumes that evolutionary patterns can change quickly and lead to independent evolutionary rates between branches. The analysis was done with the four fully sequenced *Apis* species serving as test branch against the other nine bee species from the families Apidae, Megachilidae, and Halictidae as background.

In this set of 24 divergently represented genes, four showed evidence of positive selection (Table 1), denoted by a dN/dS value > 1 and/or a *p* value < 0.05. These were as follows: *CYP9Q3, CYP6AQ1, PHGDH,* and *ACADM,* encoding, respectively, Cytochrome P450 9e2, Cytochrome P450 6AQ1 × 1, D-3 phosphoglycerate dehydrogenase, and the mitochondrial medium-chain specific acyl-CoA dehydrogenase. Among these genes, *CYP9Q3* (GB43728) and *CYP6AQ1* (GB52023) showed lower RPKM counts in the larval gonads of *A. mellifera* and *M. quadrifasciata* compared to *B. terrestris* (Fig. 2). The *PHDGH* gene (GB40503) showed low RPKM counts for *A. mellifera* queens and workers and higher counts in honey bee drones, as well as in the bumble bee and the stingless bee, but the opposite was the case for *ACADM* (GB42141), which showed higher counts in all honey bee samples and also in the stingless bee, contrasting with lower count numbers for the bumble bee.

**Table 1:** BUSTED test results for evidence of positive selection in 24 genes revealed in the hierarchical clustering analysis of honey bee, versus bumble bee and stingless bee larval gonad transcriptomes.

Gene_ID	Gene_name	Positive selection	*Apis* dN/dS	Background dN/dS	*p* value
GB40280	Pyruvate carboxylase, mitochondria, X1	−	0.0214	0.0465	0.195 ≥ .05
GB44513	Cytochrome P450 4c3	−	0.1361	0.1204	1.000 ≥ .05
GB43728	**Cytochrome P450 9e2 (CYP9Q3)**	** ++ **	**5.486**	**0.1753**	**0.000 < .05**
GB40773	Fatty acid hydroxylase domain-containing protein 2, X1	−	0.0408	0.1083	0.500 ≥ .05
GB52023	**Cytochrome P450 6AQ1 × 1 (CYP6AQ1)**	** ++ **	**0.7104**	**0.2487**	**0.000 < .05**
GB53086	Alcohol dehydrogenase class-3	−	0.1813	0.0719	0.997 ≥ .05
GB55610	Mitochondrial amidoxime reducing component 2	−	0.0915	0.2084	0.500 ≥ .05
GB51383	Probable cytochrome P450 6a14	−	0.1699	0.1826	1.000 ≥ .05
GB48195	Acyl-CoA Delta(11) desaturase	−	0.0077	0.1454	1.000 ≥ .05
GB40503	**D-3 phosphoglycerate dehydrogenase (PHDGH)**	** ++ **	**0.4861**	**0.1537**	**0.000 < .05**
GB40673	Cryl1 lambda crystallin-like protein	−	0.0318	0.0841	1.000 ≥ .05
GB42141	**Medium-chain specific acyl-coa dehydrogenase, mitochondrial (ACADM)**	** + + **	**0.3102**	**0.1209**	**0.000 < .05**
GB49347	Prostaglandin reductase 1/ cyclic AMP-dependent transcription factor ATF-6 alpha	−	0.1479	0.3188	1.000 ≥ .05
GB48308	Pyruvate dehydrogenase e1 component subunit alpha, mitochondrial, × 1	−	0.0556	0.0556	1.000 ≥ .05
GB54216	Atp-citrate synthase, × 1	−	0.0412	0.0384	1.000 ≥ .05
GB52074	6-phosphogluconate dehydrogenase, decarboxylating	−	0.0611	0.0428	1.000 ≥ .05
GB42608	Cytochrome b5	−	0.0845	0.2481	1.000 ≥ .05
GB41912	Myo-inositol 2-dehydrogenase	−	0.1248	0.1416	0.500 ≥ .05
GB49885	Cytochrome P450 6a17 (CYP6AS4)	−	0.3108	0.164	0.500 ≥ .05
GB52115	Protein CREG1	−	0.0895	0.2119	0.500 ≥ .05
GB47553	Electron transfer flavoprotein subunit alpha, mitochondrial	−	0.1423	0.0732	0.371 ≥ .05
GB49321	Sorbitol dehydrogenase	−	0.0653	0.1569	0.500 ≥ .05
GB55701	Putative aldehyde dehydrogenase family 7 member A1 homolog	−	0.2959	0.2108	0.500 ≥ .05
GB44143	Oxidative stress-induced growth inhibitor 2	−	0.1074	0.6828	0.500 ≥ .05

The four species of the genus *Apis* served as test branch against nine other bee species. Genes marked in bold showed evidence of positive selection. Gene-ID refers to the *A. mellifera* gene annotation in the Hymenoptera Genome Database. Significant values are in [bold].

**Figure 3:**
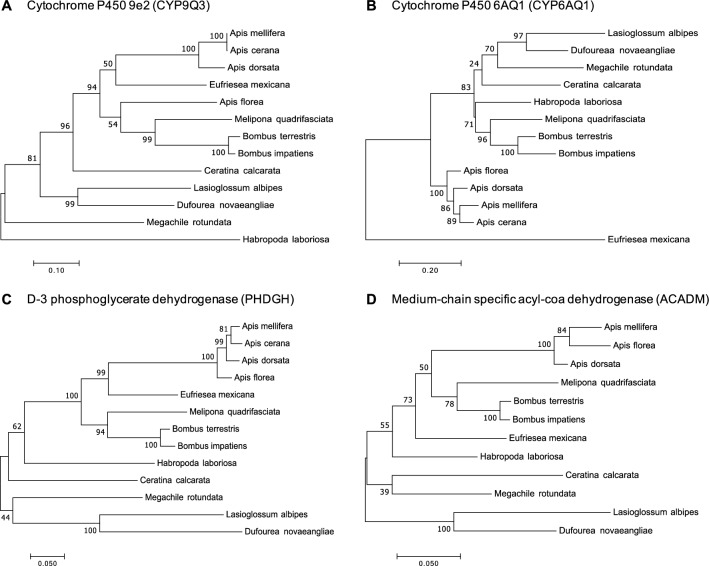
Gene trees for the four genes with evidence of positive selection with respect to bee gonad phenotype in 13 bee species. The gene trees were inferred using the Maximum Likelihood method based on the best model (Table S5), and the trees with the highest log likelihood (1000 bootstrap replicates) are shown. The trees were generated in MEGA7, with branch lengths representing the number of substitutions per site.

To obtain a more in-depth information we performed gene tree analyses for the deduced amino acid sequences of these four genes in MEGA7 software^30^, applying the models and rate substitution parameters listed in Table S5. Among the two cytochrome P450 genes, *CYP9Q3,* which had the highest index for positive selection, showed only a minor divergence with respect to the Apoidea phylogenetic tree^1,31^, and this divergence is related to the positioning of *A. florea* (Fig. 3A). In contrast, the *CYP6AQ1* gene tree showed a major divergence from the bee phylogenetic tree, with the four *Apis* species being completely separated from the representatives of the other Apoidea (Fig. 3B). The gene trees for *PHDGH* and *ACADM* (Fig. 3C and D), were largely consistent with the Apoidea phylogenetic tree.

This divergent result for the *CYP6AQ1* gene tree prompted us to take a closer look at the multiple alignments for *CYP6AQ1*. While no apparent site-restricted pattern in terms of codon changes was apparent (Fig. S3), potential functional insights were obtained through BLASTP searches against the *Drosophila melanogaster* protein annotation database, which revealed CYP6G2 and CYP6G1 as the most similar CYPs (42.94% and 44.04% identity, respectively) to the *A. mellifera* CYP6AQ1 amino acid sequence. For *A. mellifera* CYP9Q3, the most similar *D. melanogaster* CYPs were CYP9F2 and CYP6A13 (34.85% and 33.27% identity, respectively). These *Drosophila* CYPs, especially CYP6G2 and CYP6G1, are involved in interesting biological functions, such as juvenile hormone biosynthesis in the case of CYP6G2, and for CYP6G1, in DDT and imidacloprid resistance^32–35^.

## Discussion

Our study was driven by the hypothesis that the extremely polyandrous mating system in honey bees, not only in the Western honey bee, *Apis mellifera*, but in all species of the genus *Apis*^36^, and the high number of ovarioles and testioles in the gonads of both queens and drones may be evolutionarily and developmentally coupled. As this stands in contrast to all other social bees, including the highly eusocial stingless bees, which essentially have a monandrous mating system^37^, we proposed to test this by a comparative transcriptome analysis of the larval gonads of *A. mellifera* queens, workers, and drones^23^, against those of *B. terrestris* and *M. quadrifasciata* larvae as the ancestral state.

One may argue that we had missed an opportunity by pooling the gonads of the queens, workers, and males for the bumble bee and for the stingless bee, but this made sense because we had based our comparative analysis on the set of 870 *A. mellifera* genes that represented DEGs for all the three gonad contrasts (Fig. 1). Furthermore, and different from the honey bee, the larvae of the stingless bee *M. quadrifasciata* cannot be distinguished according to sex and caste, as they are all reared in brood cells of the same size and type, and in the bumble bee, *B. terrestris*, males and queens are only reared at the end of the colony cycle, separately from the worker brood.

The comparative transcriptome analysis on the set of 45 genes (Fig. 2) indicated evidence of positive (diversifying) selection in four genes that emerged as differentially represented in the gonads of the *A. mellifera* gonads compared to *M. quadrifasciata* and *B. terrestris* (Fig. 2). Two of these are cytochrome P450 genes, *CYP9Q3* and *CYP6AQ1* (Table 1). In the gene tree analyses, *CYP6AQ1* presented a configuration that set the genus *Apis* well apart from all the other Apoidea (Fig. 3), and this gene tree is highly inconsistent with the phylogenetic tree of the corbiculate bees, which has Apini as sister group to the Euglossini^1,31^. The latter are the orchid bees, whose females have a solitary or incipiently eusocial life style^38^.

Insect cytochrome P450 enzymes are involved in many metabolic and biosynthesis processes, including the synthesis of pheromones, cuticular hydrocarbons, and hormones, as well as the degradation of lipid hormones, and the detoxification of plant secondary metabolites and pesticides^33^. The annotation of cytochrome P450 genes in the first published version of the honey bee genome had indicated a significantly reduced set of orthologs for the insect GST and CYP protein clades^39^, and this finding has since been confirmed^40^. But there are exceptions, for instance the CYP6AS family, which is unique to the Hymenoptera. This family appears to have undergone an expansion in gene number, and its members have been associated with the digestion of quercetin, a flavonol compound present in nectar and pollen^41^. This is likely an adaptive trait, because quercetin acts as a competitive inhibitor of P450 enzymes, and when quercetin was fed to adult workers, these became increasingly resistant to queen pheromone and showed ovarian activation^42^. A recent phylogenetic analysis of the gene trees for a total of 481 bee P450 genes furthermore revealed that the CYP6AS clade within the CYP3 family is fast evolving and exhibits evidence of positive selection^43^. While this is mainly discussed in the context of selection pressure on the need for detoxification of secondary plant compounds, our results now indicate that this high evolutionary rate and divergence in the CYP3 family may have also been co-opted in developmental pathways that generated novelties, such as the transgressive gonad phenotype in the genus *Apis*.

Currently, experimental evidence for a reproductive system function of the *CYP6AQ1* gene is still lacking, but *CYP6AQ1* was listed as expressed in honey bee antennae^39^ and, like *CYP6AS,* it is a member of the CYP3 clade. Recently, its expression in honey bee fat body was also documented as related to aggression, and it was found down-regulated as a result of immune system activation^44^. Hence, it is quite possible that CYP6AQ1 may be involved in a signaling function that regulates gonad development and activation in bees, and through diversifying selection it may have gained a novel role associated with the transition from the ancestral monandrous mode of reproduction in social bees to polyandry in the genus *Apis.* A current difficulty still is to understand how the evidenced positive selection may have resulted in functional changes in the honey bee CYP6AQ1 protein. In the CLUSTALW alignment of the bee orthologs of CYP6AQ1 (Fig. S3), the non-synonymous substitutions appeared spread out across the entire CDS, and there is currently no information available on the location and structure of the enzyme’s catalytic site. Most cytochrome P450 enzymes have broad catalytic properties^34^, and detailed mappings on substrate recognition sites have so far only been done for a handful of insect cytochrome P450 enzymes, such as certain CYP6B sequences of lepidopterans^45^ and for the housefly CYP6A1^46^.

Our BLASTP searches against the *Drosophila* genome identified CYP6G1 and CYP6G2 as the most similar ones to the honey bee CYP6AQ1, while CYP9F2 and CYP6A13 were the most similar ones to the honey bee CYP9Q3. In the *Drosophila* genome, *CYP6G1* and *CYP6G2* map right next to each other^47^, possibly resulting from a duplication, but they are involved into very different functions. While *CYP6G1* has long been identified as a gene related to insecticide resistance, especially against DDT and the neonicotinoid imidacloprid^48^, *CYP6G2* is highly expressed in the ring gland of *Drosophila* larvae, where it is considered to be involved in juvenile hormone (JH) biosynthesis^35^. These functional annotations in *Drosophila* are of interest with respect to honey bee biology. On the one hand, JH is a major driver in honey bee caste development and also for the behavioral maturation of adult workers^49^. On the other hand, imidacloprid is a neonicotinoid that is strongly associated with colony losses^50^, including sublethal effects on the learning and memory of honey bee workers, which affect the homing of foragers as they return from nectar and pollen resources. Furthermore, JH has recently been shown to play a critical role in the brain-reproduction energetics tradeoff that distinguishes the honey bees from the bumble bees^51^.

JH is a fundamental hormone in the insects’ life cycles, orchestrating molting, metamorphosis, and reproduction, and it is a key driver for the adaptive expression of characters, including the castes of social insects^52^. Together with our results that indicate positive selection acting on the *CYP6AQ1* gene in the *Apis* lineage, selection on JH signaling-related genes in the evolution of sociality has recently also been demonstrated in a comprehensive comparative genomics study in halictid bees^53^. These authors looked for evidence of positive selection in genes associated with lineages that have independently made the transition from a solitary to a eusocial lifestyle, versus relaxed selection in those lineages where the females reverted from eusociality to secondarily become solitary nesting. Under this premise, four genes showed such contrasting evidence. Two of these, apolipoprotein and hexamerin 110, are abundant insect hemolymph proteins related to JH binding and transport.

In conclusion, the fact that we and others^53^ could identify evidence of positive (diversifying) or relaxed selection in genes or gene families that are related to important transitions in the evolution of insect sociality shows the strength of comparative genomics or transcriptomics when performed under a well-defined phylogenetic background. Though not all these genes may be directly associated with important functions, such as JH signaling, they can heuristically guide future research efforts on how natural selection may have shaped developmental and behavioral plasticity^54^, which is key to the queen-worker polyphenism in social insects.

## Methods

### Sample preparation

Stingless bee larvae (*M. quadrifasciata*) were obtained during the Southern summer season (January/February) from brood combs of three hives kept in the Experimental Apiary of the University of São Paulo in Ribeirão Preto, Brazil. The larval stage equivalent to the fifth-instar early feeding stage (L5F1) of honey bees used in our prior study^23^ is the early third instar (L3.1) of *Melipona* bees^55^. Bumble bee (*B. terrestris*) larvae were obtained during the Northern summer season (June/July) from three commercial Koppert pollination hives (Behr Bestäubung Biologischer Pflanzenschutz Welle, Germany). These hives were kept in the Baden-Württemberg State Bee Research Institute at the University of Hohenheim, Stuttgart, Germany. The developmental schedule listed in^56^ was used for the identification and dissection of third instar *B. terrestris* larvae, which is the stage corresponding to the L5F1 stage of *A. mellifera,* for which RNA was extracted simultaneously and at the location^23^.

The larval gonads of all three species, *A. mellifera*^23^, *M. quadrifasciata*, and *B. terrestris*, were dissected in sterile insect saline and immediately transferred to TRIzol reagent (Life Technologies), and the manufacturer’s protocol for the use with small tissue quantities was used for RNA extraction: TRIzol (500 μL), chloroform (100 μL), glycogen (5 μg), and overnight precipitation (− 80 °C) with isopropanol (250 μL). After solvent evaporation, the RNA was resuspended in 20 μL RNase-free water. The number of gonads composing each *A. mellifera* sample is reported in^23^. For *B. terrestris*, each of the three biological samples consisted of 40 larval gonad pairs, and for *M. quadrifasciata,* the number of gonad pairs was 50 for each of the three samples. Sample quality control, library preparation, and high-throughput sequencing protocols were all performed simultaneously by CeGaT GmbH (Tübingen, Germany), as previously described^23^. Paired-end sequencing was carried out on a NovaSeq6000 (Ilumina) platform, with read lengths of 2 × 100 bp and an output of 6 Gb per sample.

### RNA-seq bioinformatics analysis

The initial RNA sequencing data analysis was done using the Nextflow-based RNA-Seq pipeline release 1.2 (https://github.com/nf-core/rnaseq). It included the MultiQC v1.6 program^57^ and FASTQC v0.11.8 to determine FASTQ file quality, and after removal of low-quality reads, adapter sequences were trimmed with Trim Galore 0.5.0. The raw trimmed sequences of each of the three *B. terrestris* and *M. quadrifasciata* larval gonad RNA-seq libraries were submitted to NCBI (SRA, accession number: *B. terrestris* SRX8487631; *M. quadrifasciata* SRX8487632).

Next, using the HISAT2 v2.1.0 aligner^58^, the reads that had passed the quality control were mapped to the respective genomes of *Bombus terrestris* (fasta: Bombus_terrestris.Bter_1.0.dna.toplevel.fa, gff3: Bombus_terrestris.Bter_1.0.40.gff3), and the *Melipona quadrifasciata* genome (fasta: GCA_001276565.1_ASM127656v1_genomic.fna, gtf: Melipona_quadrifasciata_v1.1_on_GCA_001276565.1_ASM127656v1_complete_mar2_2017.gtf). An evaluation of the mapped RNA-seq reads was done with RSeQC v2.6.4^59^, and for read counting of the features (e.g., genes), FeatureCounts v1.6.2^60^ was used. The read counts were then normalized to RPKM to remove potential technical bias in the RNA-seq data, such as depth of sequencing and gene length. All programs were used with default settings for mismatch allowance.

### Hierarchical clustering analysis

The starting point of the analysis were the 870 DEGs (Fig. 1) of the honey bee larval gonads, based on the previously published RNA-seq data set^23^. The focal genes for the comparison across the three bee species were defined by a Gene Ontology (GO) enrichment analysis on the 870 *A. mellifera* larval gonad DEGs performed using Blast2GO implemented in OmicsBox (biobam, Valencia, Spain). Enrichment significance was assessed using Fisher’s Exact Test. This resulted in a set of 45 genes, for which the *B. terrestris* orthologs were retrieved from GenBank using the NCBI BLASTP tool. For *M. quadrifasciata,* the respective BLASTP searches were done in the Hymenoptera Genome Database (hymenopteragenome.org)^27^. Ortholog tables with the respective gene_id (available in HymenopteraMine v1.4; http://hymenopteragenome.org/hymenopteramine/)^61^ were used to find and manually confirm the corresponding gene_ids. Their orthology status was confirmed by reciprocal BLASTP searches against the honey bee genome.

Using the respective gene_id, the RPKM counts for each gene were retrieved from the 15 transcriptomes (9 for *A. mellifera* and 3 each for *B. terrestris* and *M. quadrifasciata*). A principal component analysis and a hierarchical cluster analysis on the RPKM counts were performed in JMP Genomics 12.2.0 software (SAS).

### Analysis of positive selection in candidate genes

For the 24 genes that showed clear contrasts for the honey bee gonad transcriptomes against those of the bumble bee and the stingless bee in the hierarchical clustering analysis, BLAST tools (BLASTP and then BLASTN) were used to extract the corresponding nucleotide sequences (CDS) of their orthologs from the genomes of 13 bee species deposited in GenBank and the Hymenoptera Genome Database^27^ (Table S4). These included four species of the genus *Apis* (*A. mellifera, A. cerana, A. dorsata,* and *A. florea*)*,* two bumble bee species *(Bombus terrestris* and *B. impatiens),* the stingless bee *Melipona quadrifasciata,* the orchid bee *Eufriesea mexicana,* the carpenter bee *Ceratina calcarata,* and the anthophorid *Habropoda laboriosa.* These 10 species all belong to the family Apidae. In addition, we included *Megachile rotundata* (Megachilidae) and two Halictidae*, Lasioglossum albipes* and *Dufourea novaeangliae*. The respective nucleotide sequences were aligned using ClustalW and visualized in Aliview^62^. The software geneious (https://www.geneious.com) was used to convert the nucleotide sequences into consensus amino acid sequences, and a codon alignment analysis was performed using Pal2Nal (http://www.bork.embl.de/pal2nal/index.cgi?example=Yes#RunP2N).

To reveal whether genes or specific sites may have experienced episodes of positive selection, the codon alignment was used as input to an evolutionary test performed in BUSTED^29^, implemented in the DataMonkey server (https://datamonkey.org/busted). BUSTED checks for synonymous and non-synonymous substitutions and calculates the dN/dS value for each gene. Additionally, it computes the dN/dS value for the respective gene within each species and calculates a *p* value for the gene in the comparison between the test branch versus the background branches. With this, a dN/dS-value is attributed to each gene, and a result for evidence of selection (*p* < 0.05) is given. This avoids relying on dN/dS-values alone, as these have been shown to have limited power in detecting positive selection^63^. In our analysis, the four species of the genus *Apis* served as test branch against nine other bee species.

Evolutionary trees were generated for the four genes with evidence of positive selection using MEGA7 software^30^. Modeltest implemented in MEGA was used to identify the evolutionary model that best described the amino acid substitution pattern for each data set. The model with the lowest BIC scores (Bayesian Information Criterion) was the used for the construction of unrooted trees based on Maximum Likelihood. Models, BIC scores, as well as the G+ and I+ values used for gene tree construction are listed in Table S5. Bootstrap analyses with 1000 replicates were run for each tree.

### Ethical approval and consent to participate

Not applicable. As no chordate species were used, the Committee for the Use of Animals in Experiments (CEUA) of FMRP-USP declared the study protocol as exempt from regulation.

## Supplementary Information

Supplementary Information.

## Data Availability

The raw trimmed sequences of the RNA-seq libraries were deposited as: NCBI Bioproject ID *A. mellifera* PRJNA636804, *B. terrestris* PRJNA636805, *M. quadrifasciata* PRJNA636806. They were submitted to the Sequence Read Archive (SRA, accession number: *A. mellifera*: Worker SRR11940311; Queen SRR11940310; Drone SRR11940309; *B. terrestris* SRR11943140; *M. quadrifasciata* SRR11943141).
