# Supplementary material for: Positive selection in cytochrome P450 genes is associated with gonad phenotype and mating strategy in social bees
Source: Sci Rep. 2023 Apr 11;13:5921. doi: 10.1038/s41598-023-32898-6 (PMC10090045; doi:10.1038/s41598-023-32898-6)
Supplement: Supplementary file 1 — Supplementary Information. [file 41598_2023_32898_MOESM1_ESM.docx]

**Supplementary Material**

**Supplementary Tables**

**Table S1.** Gene Ontology (GO) enrichment analysis for the 870 DEGs shared in all three contrasts of *A. mellifera* gonads.

| **GO_ID** | **GO_name** | **Category^a^** | **FDR^b^** | ***p*-value** | **n_test^c^** | **n_reference^d^** |
| --- | --- | --- | --- | --- | --- | --- |
| GO:0016705 | Oxidoreductase activity, acting on paired donors, with incorporation or reduction of molecular oxygen | MF | 7.8 E-8 | 8.6 E-11 | 24 | 65 |
| GO:0055114 | Oxidation-reduction process | BP | 4.6 E-7 | 5.9 E-10 | 63 | 423 |
| GO:0005506 | Iron ion binding | MF | 1.9 E-6 | 2.8 E-9 | 21 | 60 |
| GO:0004497 | Monooxygenase activity | MF | 2 E-6 | 4 E-9 | 18 | 43 |
| GO:0005524 | ATP binding | MF | 2.3 E-5 | 9.1 E-8 | 78 | 666 |
| GO:0020037 | Heme binding | MF | 4.3 E-5 | 2 E-7 | 20 | 73 |
| GO:0006508 | Proteolysis | BP | 1.5 E-4 | 8.1 E-7 | 45 | 321 |
| GO:0005515 | Protein binding | MF | 5.8 E-4 | 3.8 E-6 | 150 | 1712 |
| GO:0016301 | Kinase activity | MF | 8.2 E-4 | 5.9 E-6 | 40 | 293 |
| GO:0016773 | Phosphotransferase activity, alcohol group as acceptor | MF | 3.9 E-3 | 2.6 E-5 | 35 | 263 |
| GO:0016310 | Phosphorylation | BP | 5.2 E-3 | 5.4 E-5 | 41 | 338 |
| GO:0016021 | Integral component of membrane | CC | 5.8 E-3 | 6.2 E-5 | 200 | 2.551 |
| GO:0015020 | Glucuronosyltransferase activity | MF | 1.1 E-2 | 1.2 E-4 | 5 | 5 |
| GO:0022857 | Transmembrane transporter activity | MF | 1.3 E-2 | 1.5 E-4 | 43 | 380 |
| GO:0042593 | Glucose homeostasis | BP | 1.5 E-2 | 1.8 E-4 | 3 | 0 |
| GO:0019752 | Carboxylic acid metabolic process | BP | 1.6 E-2 | 1.9 E-4 | 26 | 187 |
| GO:0051169 | Nuclear transport | BP | 1.7 E-2 | 2.1 E-4 | 9 | 29 |
| GO:0008235 | Metalloexopeptidase activity | MF | 2.1 E-2 | 2.7 E-4 | 7 | 17 |
| GO:0055085 | Transmembrane transport | BP | 3 E-2 | 3.9 E-4 | 48 | 462 |
| GO:0003774 | Motor activity | MF | 3.6 E-2 | 4.7 E-4 | 13 | 66 |
| GO:0048029 | Monosaccharide binding | MF | 3.8 E-2 | 5.1 E-4 | 5 | 8 |

The analysis was performed using Fisher’s exact test on Blast2GO, implemented in OmicsBox. a. Type of the GO_ID, as Biological Process (BP), Molecular Function (MF), or Cell Component (CC). b. Significance represented by FDR and *p*-value. c. n_test represents the number of DEGs within each category. d. n_reference represents the total number of genes representing the respective category in the analysis platform.

**Table S2.** The 45 honey bee larval gonad DEGs represented in the enriched GO categories.

| **Gene_ID^a^** | **Gene name** | **NCBI_ID^b^** |
| --- | --- | --- |
| GB40280 | Pyruvate carboxylase, mitochondria, X1 | XM_006568043.3 |
| GB40284 | Cytochrome P450 6a14 (CYP6AS10) | XM_016915831.2 |
| GB40503 | D-3 phosphoglycerate dehydrogenase | XM_006559535.3 |
| GB40517 | Uncharacterized protein CG3556 | XM_006559578.3 |
| GB40673 | Cryl1 lambda crystallin-like protein | NM_001135735.1 |
| GB40773 | Fatty acid hydroxylase domain-containing protein 2, X1 | XM_016913284.2 |
| GB41912 | Myo-inositol 2-dehydrogenase | XM_624405.6 |
| GB42052 | Estradiol 17-beta-dehydrogenase 2 | XM_016912293.2 |
| GB42141 | Medium-chain specific acyl-coa dehydrogenase, mitochondrial | XM_392111.7 |
| GB42239 | Senecionine N-oxygenase | XM_006571198.3 |
| GB42608 | Cytochrome b5 | XM_001120801.4 |
| GB43006 | Glucose dehydrogenase (GMCOX13) | XM_392145.7 |
| GB43713 | Cytochrome P450 9e2 (CYP9Q1) | XM_006562301.3 |
| GB43727 | Cytochrome P450 9e2 (CYP9Q2) | XM_392000.7 |
| GB43728 | Cytochrome P450 9e2 (CYP9Q3) | XM_006562300.3 |
| GB44143 | Oxidative stress-induced growth inhibitor 2 | XM_006567172.3 |
| GB44513 | Cytochrome P450 4c3 | XM_016912202.2 |
| GB45193 | Glutaryl-CoA dehydrogenase, mitochondrial (Gdch) | XM_393736.6 |
| GB45609 | Flavin-containing monooxygenase FMO GS-OX-like 4 | XM_001122155.5 |
| GB45973 | Aromatic-L-amino-acid decarboxylase (Ddc) | XM_394115.7 |
| GB46510 | Poly [ADP-ribose] polymerase (Parp) | XM_624474.6 |
| GB46579 | Glucose-6-phosphate 1-dehydrogenase | XM_006564722.3 |
| GB46737 | Hydroxyacid oxidase 1 | XM_625146.6 |
| GB46814 | Cytochrome P450 6k1 (CYP6BE1) | XM_016917348.2 |
| GB47503 | Delta-1-pyrroline-5-carboxylate synthase | XM_026446211.1 |
| GB47553 | Electron transfer flavoprotein subunit alpha, mitochondrial | XM_624099.5 |
| GB48195 | Acyl-CoA Delta(11) desaturase | XM_026440107.1 |
| GB48308 | Pyruvate dehydrogenase e1 component subunit alpha, mitochondrial, x1 | XM_006572095.3 |
| GB49321 | Sorbitol dehydrogenase | XM_393651.6 |
| GB49347 | Prostaglandin reductase 1/ cyclic AMP-dependent transcription factor ATF-6 alpha | XM_394852.7 |
| GB49885 | Cytochrome P450 6a17 (CYP6AS4) | XM_395671.6 |
| GB50076 | Spermine oxidase | XM_396922.7 |
| GB50627 | Putative fatty acyl-CoA reductase | NM_001190920.1 |
| GB50678 | Prolyl 3-hydroxylase OGFOD1 | XM_003249424.4 |
| GB50971 | Dihydropyrimidine dehydrogenase [NADP(+)] | XM_016914269.2 |
| GB51238 | Acyl-CoA Delta(11) desaturase | XM_623922.6 |
| GB51383 | Probable cytochrome P450 6a14 (CYP6AR1) | XM_623359.6 |
| GB52023 | Cytochrome P450 6AQ1 x1 (CYP6AQ1) | XM_016915338.2 |
| GB52074 | 6-phosphogluconate dehydrogenase, decarboxylating | XM_006566923.2 |
| GB52115 | Protein CREG1 | XM_026444487.1 |
| GB53086 | Alcohol dehydrogenase class-3 | XM_393266.6 |
| GB54216 | Atp-citrate synthase, x1 | XM_623080.5 |
| GB55610 | Mitochondrial amidoxime reducing component 2 | XM_026438947.1 |
| GB55638 | Tryptophan 2,3-dioxygenase | XM_006568986.3 |
| GB55701 | Putative aldehyde dehydrogenase family 7 member A1 homolog | XM_394614.7 |

a. Gene-ID is the code for honey bee genes in the Hymenoptera database; b. NCBI-ID is the respective code in GenBank.

**Table S3.** The 24 honey bee larval gonad DEGs showing divergent RPKM levels in the transcriptomes of the three bee species (Figure 2).

| **Gene_ID** | ***Apis mellifera* taxid:7460** | **Gene_name** | **LG** | **Exon count** | **Location** |
| --- | --- | --- | --- | --- | --- |
| GB40280 | XM_006568043.3 | Pyruvate carboxylase, mitochondria, X1 | LG13 | 14 | NC_007082.3 (9852769..9882453) |
| GB44513 | XM_016912202.2 | Cytochrome P450 4c3 | LG5 | 8 | NC_007074.3 (10336742..10347273) |
| GB43728 | XM_006562300.3 | Cytochrome P450 9e2 (CYP9Q3) | LG14 | 1 | NC_007083.3 (5458368..5460082) |
| GB40773 | XM_016913284.2 | Fatty acid hydroxylase domain-containing protein 2, X1 | LG1 | 8 | NC_007070.3 (6139732..6150435) |
| GB52023 | XM_016915338.2 | Cytochrome P450 6AQ1 x1 (CYP6AQ1) | LG12 | 7 | NC_007081.3 (9418194..9421737) |
| GB53086 | XM_393266.6 | Alcohol dehydrogenase class-3 | LG4 | 7 | NC_007073.3 (2616107..2618415) |
| GB55610 | XM_026438947.1 | Mitochondrial amidoxime reducing component 2 | LG2 | 5 | NC_007071.3 (13479351..13487242) |
| GB51383 | XM_623359.6 | Probable cytochrome P450 6a14 | LG5 | 5 | NC_007074.3 (3901710..3905058) |
| GB48195 | XM_026440107.1 | Acyl-CoA Delta(11) desaturase | LG4 | 7 | NC_037641.1 (2193663..2223709) |
| GB40503 | XM_006559535.3 | D-3 phosphoglycerate dehydrogenase | LG8 | 7 | NC_007077.3 (2814201..2817692) |
| GB40673 | NM_001135735.1 | Cryl1 lambda crystallin-like protein | LG12 | 6 | NC_007081.3 (7138299..7140695) |
| GB42141 | XM_392111.7 | Medium-chain specific acyl-coa dehydrogenase, mitochondrial | LG1 | 10 | NC_007070.3 (1341759..1344255) |
| GB49347 | XM_394852.7 | Prostaglandin reductase 1/ cyclic AMP-dependent transcription factor ATF-6 alpha | LG12 | 4 | NC_007081.3 (8879422..8883253) |
| GB48308 | XM_006572095.3 | Pyruvate dehydrogenase e1 component subunit alpha, mitochondrial, x1 | LG12 | 7 | NC_037649.1 (3090091..3092610) |
| GB54216 | XM_623080.5 | Atp-citrate synthase, x1 | LG14 | 12 | NC_037651.1 (6574437..6583128) |
| GB52074 | XM_006566923.2 | 6-phosphogluconate dehydrogenase, decarboxylating | LG12 | 5 | NC_007081.3 (10491274..10493871) |
| GB42608 | XM_001120801.4 | Cytochrome b5 | LG9 | 2 | NC_007078.3 (7406515..7410127,) |
| GB41912 | XM_624405.6 | Myo-inositol 2-dehydrogenase | LG8 | 4 | NC_007077.3 (7465167..7468787) |
| GB49885 | XM_395671.6 | Cytochrome P450 6a17 (CYP6AS4) | LG13 | 5 | NC_007082.3 (9573856..9576680) |
| GB52115 | XM_026444487.1 | Protein CREG1 | LG12 | 5 | NC_007081.3 (11712163..11716382) |
| GB47553 | XM_624099.5 | Electron transfer flavoprotein subunit alpha, mitochondrial | LG16 | 4 | NC_007085.3 (4858916..4860776,) |
| GB49321 | XM_393651.6 | Sorbitol dehydrogenase | LG12 | 6 | NC_007081.3 (8522396..8529126) |
| GB55701 | XM_394614.7 | Putative aldehyde dehydrogenase family 7 member A1 homolog | LG6 | 9 | NC_007075.3 (18340965..18343920) |
| GB44143 | XM_006567172.3 | Oxidative stress-induced growth inhibitor 2 | LG4 | 8 | NC_007073.3 (9700334..9707954) |

a. Gene-ID refers to the *A. mellifera* gene annotation in the Hymenoptera Genome Database.

b. Linkage group and location at Amel_4.5 assembly.

**Table S4.** Ortholog codes for the 24 candidate DEGs in the 13 bee species used in the evolutionary analysis. BLAST tools were used to find the orthologs and extract their respective nucleotide sequences (CDS). Numbers below species names refer to taxon-ID in NCBI.

| **Gene ID** | **Gene_name** | ***Apis mellifera* 7460** | ***Apis cerana* 7461** | ***Apis dorsata* 7462** | ***Apis florea* 7463** | ***Bombus terrestris* 30195** | ***Bombus impatiens* 132113** | ***Melipona quadrifasciata* 166423** | ***Eufriesea mexicana* 516756** | ***Habropoda laboriosa* 597456** | ***Dufourea novaeangliae* 178035** | ***Megachile rotundata* 143995** | ***Lasioglossum albipes***  **88501** | ***Ceratina calcarata* 156304** |
| --- | --- | --- | --- | --- | --- | --- | --- | --- | --- | --- | --- | --- | --- | --- |
| GB40280 | Pyruvate carboxylase, mitochondria, X1 | XM_006568043.3 | XM_017066635.2 | XM_006614198.1 | XM_003694456.2 | XM_003400089.3 | XM_003494382.3 | MQUA17286 | XM_017902311.1 | XM_017935815.1 | XM_015576672.1 | XM_003704235.2 | LALB15261 | XM_026811033.1 |
| GB44513 | Cytochrome P450 4c3 | XM_016912202.2 | XM_017056855.1 | XM_006618564.1 | XM_003696581.2 | XM_003401923.3 | XM_012391452.2 | MQUA24885 | XM_017905728.1 | XM_017938115.1 | XM_015576418.1 | XM_003699504.2 | LALB13061 | - |
| GB43728 | Cytochrome P450 9e2 (CYP9Q3) | XM_006562300.3 | KT697624.1 | XM_006612959.1 | XM_012492383.1 | XM_003393328.3 | XM_003486002.3 | MQUA06607 | XM_017903151.1 | XM_017935066.1 | XM_015583533.1 | XM_003705443.2 | Lalb_07001 | XM_018031669.2 |
| GB40773 | Fatty acid hydroxylase domain-containing protein 2, X1 | XM_016913284.2 | XM_017052140.2 | XM_006610548.1 | XM_012494674.1 | XM_003396159.3 | XM_012386750.2 | MQUA18412 | XM_017904753.1 | XM_017942824.1 | XM_015578517.1 | XM_012292663.1 | LALB15462 | XM_018024265.2 |
| GB52023 | Cytochrome P450 6AQ1 x1 (CYP6AQ1) | XM_016915338.2 | XM_017066161.2 | XM_006610792.1 | XM_003697371.2 | XM_012314843.2 | XM_012389238.2 | MQUA23909 | XM_017911594.1 | XM_017935936.1 | XM_015581499.1 | XM_003701297.2 | Lalb_06384 | NM_001365339.1 |
| GB53086 | Alcohol dehydrogenase class-3 | XM_393266.6 | XM_017065885.2 | XM_006612160.1 | XM_003691701.2 | XM_003395043.3 | XM_003491213.3 | MQUA18940 | XM_017903070.1 | XM_017941666.1 | XM_015575191.1 | XM_003702991.2 | LALB16703 | XM_018022310.2 |
| GB55610 | Mitochondrial amidoxime reducing component 2 | XM_026438947.1 | XM_028669151.1 | XM_006623307.1 | XM_003696802.2 | XM_003393974.3 | XM_003490609.3 | MQUA13461 | XM_017910972.1 | XM_017934605.1 | XM_015576313.1 | XM_003702196.2 | Lalb_03679 | - |
| GB51383 | Probable cytochrome P450 6a14 | XM_623359.6 | XM_017062424.2 | XM_006612760.1 | XM_003696756.2 | XM_012315467.2 | NM_001365859.1 | MQUA12175 | XM_017902542.1 | XM_017940213.1 | XM_015579289.1 | XM_012287682.1 | LALB12132 | XM_018036275.2 |
| GB48195 | Acyl-CoA Delta(11) desaturase | XM_026440107.1 | XM_017060238.2 | XM_006624858.1 | XM_012487408.1 | NM_001280889.2 | XM_012390094.2 | MQUA11233 | EMEX13872 | XM_017941741.1 | XM_015576293.1 | XM_012286735.1 | LALB15743 | XM_026817820.1 |
| GB40503 | D-3 phosphoglycerate dehydrogenase | XM_006559535.3 | XM_017066905.2 | XM_006607206.1 | XM_012484957.1 | XM_003401794.3 | XM_003490218.3 | MQUA10541 | XM_017909030.1 | XM_017931950.1 | XM_015574957.1 | XM_003701090.2 | Lalb_00602 | XM_026817861.1 |
| GB40673 | Cryl1 lambda crystallin-like protein | NM_001135735.1 | XM_017053045.1 | XM_006608876.1 | XM_003698325.2 | XM_003399471.3 | XM_003484443.2 | MQUA23941 | XM_017897953.1 | XM_017936015.1 | XM_015583181.1 | XM_003700138.2 | LALB20357 | XM_018032972.2 |
| GB42141 | Medium-chain specific acyl-coa dehydrogenase, mitochondrial | XM_392111.7 | - | XM_006620170.1 | XM_003690741.2 | XM_012310244.2 | XM_024366058.1 | MQUA15713 | XM_017908366.1 | XM_017936572.1 | XM_015574444.1 | XM_003702154.2 | LALB11532 | XM_018024942.2 |
| GB49347 | Prostaglandin reductase 1/ cyclic AMP-dependent transcription factor ATF-6 alpha | XM_394852.7 | XM_017058555.2 | XM_006615541.1 | XM_003698193.2 | XM_020866046.1 | XM_003484447.2 | MQUA14635 | EMEX11529 | XM_017936934.1 | XM_015573855.1 | XM_003701279.2 | LALB10979 | XM_018033035.2 |
| **Gene ID** | **Gene_name** | ***Apis mellifera* 7460** | ***Apis cerana* 7461** | ***Apis dorsata* 7462** | ***Apis florea* 7463** | ***Bombus terrestris* 30195** | ***Bombus impatiens* 132113** | ***Melipona quadrifasciata* 166423** | ***Eufriesea mexicana* 516756** | ***Habropoda laboriosa* 597456** | ***Dufourea novaeangliae* 178035** | ***Megachile rotundata* 143995** | ***Lasioglossum albipes***  **88501** | ***Ceratina calcarata* 156304** |
| GB48308 | Pyruvate dehydrogenase e1 component subunit alpha, mitochondrial, x1 | XM_006572095.3 | XM_017049639.2 | XM_006610843.1 | XM_003698321.2 | XM_003399734.2 | XM_003493664.3 | MQUA14691 | XM_017906668.1 | XM_017939334.1 | XM_015577191.1 | XM_003704090.2 | LALB12784 | XM_026816863.1 |
| GB54216 | Atp-citrate synthase, x1 | XM_623080.5 | XM_017061130.2 | XM_006616643.1 | XM_003694391.2 | XM_020866548.1 | XM_012388391.1 | MQUA10224 | XM_017903891.1 | XM_017942215.1 | XM_015578677.1 | XM_003708271.2 | LALB12743 | XM_018029212.2 |
| GB52074 | 6-phosphogluconate dehydrogenase, decarboxylating | XM_006566923.2 | XM_017057632.2 | XM_006613427.1 | XM_012491683.1 | XM_003399908.3 | XM_003494045.3 | MQUA11429 | XM_017908649.1 | XM_017935845.1 | XM_015576773.1 | XM_012279262.1 | LALB11767 | XM_018019857.2 |
| GB42608 | Cytochrome b5 | XM_001120801.4 | XM_017058865.2 | XM_006610059.1 | XM_003696759.2 | XM_003397758.2 | XM_003488786.2 | MQUA10665 | XM_017899565.1 | XM_017933729.1 | XM_015580902.1 | XM_012296508.1 | LALB16454 | XM_026813792.1 |
| GB41912 | Myo-inositol 2-dehydrogenase | XM_624405.6 | XM_017054478.2 | XM_006618428.2 | XM_003694017.3 | XM_003403161.3 | XM_003486318.3 | MQUA24985 | EMEX18581 | HLAB15984 | XM_015574927.1 | XM_003701059.2 | LALB10589 | XM_018021810.2 |
| GB49885 | Cytochrome P450 6a17 (CYP6AS4) | XM_395671.6 | XM_017066590.2 | XM_006614202.2 | XM_003694514.3 | XM_012315465.2 | XM_024371939.1 | MQUA12174 | EMEX13069 | HLAB19465 | XM_015579288.1 | XM_003704230.2 | LALB19295 | XM_026817800.1 |
| GB52115 | Protein CREG1 | XM_026444487.1 | XM_017058494.2 | XM_006611956.2 | XM_003695219.3 | XM_003399656.3 | XM_003493724.3 | MQUA18426 | EMEX18393 | XM_017939564.1 | XM_015583180.1 | XM_003701282.2 | LALB19754 | XM_018022070.2 |
| GB47553 | Electron transfer flavoprotein subunit alpha, mitochondrial | XM_624099.5 | XM_017060680.1 | XM_006620448.2 | XM_012492535.2 | XM_003402112.3 | XM_003493493.3 | MQUA17637 | XM_017898089.1 | XM_017937281.1 | XM_015572987.1 | XM_003700381.2 | LALB17481 | XM_018031152.1 |
| GB49321 | Sorbitol dehydrogenase | XM_393651.6 | XM_017062592.2 | XM_006615609.1 | XM_003695160.3 | XM_003399817.3 | XM_003486766.2 | MQUA16805 | XM_017900756.1 | XM_017932066.1 | XM_015576636.1 | XM_012297043.1 | LALB10968 | XM_026818941.1 |
| GB55701 | Putative aldehyde dehydrogenase family 7 member A1 homolog | XM_394614.7 | XM_017058799.2 | XM_006616822.2 | XM_003692417.3 | XM_012311836.2 | XM_003486156.3 | MQUA22742 | XM_017907409.1 | XM_017933194.1 | XM_015579761.1 | XM_003703454.2 | LALB20469 | XM_018029201.2 |
| GB44143 | Oxidative stress-induced growth inhibitor 2 | XM_006567172.3 | XM_017063818.2 | XM_006617369.2 | XM_012487274.2 | XM_003395202.3 | XM_012384198.2 | MQUA11057 | XM_017901663.1 | HLAB19780 | XM_015575240.1 | XM_012292011.1 | LALB15024 | XM_018038139.2 |

**Table S5.** Models used for gene tree construction in MEGA7. Models with the lowest BIC scores (Bayesian Information Criterion) were considered to best describe the substitution pattern.

| **Gene_ID** | **Gene_name** | **Model** | **BIC** | **I+** | **G+** |
| --- | --- | --- | --- | --- | --- |
| GB43728 | Cytochrome P450 9e2 (CYP9Q3) | GTR+G | 31,528.006 | n/a | 0.94 |
| GB52023 | Cytochrome P450 6AQ1 x1 (CYP6AQ1) | GTR+G+I | 28,452.138 | 0.14 | 1.41 |
| GB40503 | D-3 phosphoglycerate dehydrogenase | K2+G+I | 20,931.974 | 0.33 | 1.48 |
| GB42141 | Medium-chain specific acyl-coa dehydrogenase, mitochondrial | TN93+G | 13,529.939 | n/a | 0.42 |

**Supplementary Figures**

**Figure S1.** Hierarchical clustering analysis of the 870 honey bee DEGs against their respective orthologs in *Bombus terrestris.* The comparison was done for the RPKM counts of 806 clear orthologs between *A. mellifera* and *B. terrestris*. Blue represents genes with low RPKM counts, while red represents those with high RPKM counts.

**Figure S2.** Principal component analysis (PCA) of the RNA-seq data for *Apis mellifera, Bombus terrestris,* and *Melipona quadrifasciata* larval gonads*.* The two first principal components, PC1 and PC2, explain 96,25% of the total sample variance. The PCA on covariances was performed in JMP Genomics 12.2.0 with factor 3.

**Figure S3.** CLUSTALW alignment of the bee orthologs of *Cytochrome P450 6AQ1* x1 (*CYP6AQ1*
